# A Meta-Analysis of Gender Differences in e-Learners' Self-Efficacy, Satisfaction, Motivation, Attitude, and Performance Across the World

**DOI:** 10.3389/fpsyg.2022.897327

**Published:** 2022-05-18

**Authors:** Zhonggen Yu, Xinjie Deng

**Affiliations:** Faculty of Foreign Studies, Beijing Language and Culture University, Beijing, China

**Keywords:** gender differences, self-efficacy, satisfaction, motivation, attitude, performance

## Abstract

E-learning has gained popularity since the outbreak of COVID-19. This study aims to identify gender differences in e-learners' self-efficacy, satisfaction, motivation, attitude, and performance across the world. Through a meta-analysis and systematic review, this study concludes that there are generally no significant gender differences in e-learning outcomes except in a few countries. Females significantly outperformed males in Spain and the UK. In Austria, India, and mixed countries (Chile and Spain), females hold significantly more positive attitudes toward e-learning than males. In the USA, females present significantly higher self-efficacy than males. Future research into the gender issue in e-learning across the world may adopt cross-disciplinary research methods except for a meta-analysis.

## Introduction

With the rapid development of science and technology, the new century has been witnessing growing self-efficacy, satisfaction, motivation, attitude, and performance among e-learners (Thompson et al., 2002). This significant growth has also highlighted the necessity to examine the influence of gender differences on e-learners' self-efficacy, satisfaction, motivation, attitude, and performance across the world.

Self-efficacy in e-learning, positively influencing e-learning effectiveness (Hsu and Chiu, 2004), was operationally defined as the individual evaluation of the e-learning experience and the individual ability to complete a given e-learning task (Torkzadeh and Van Dyke, 2002). Previous studies reported significant differences in e-learning self-efficacy (e.g., Chen and Tsai, 2007). Presence of males could lead to significantly higher self-efficacy than females (Baylor and Kim, 2004). Learners with higher self-efficacy could be able to obtain more knowledge by focusing on online resources, perform better by spending more time and be more motivated to engage in e-learning than those with lower self-efficacy (Pituch and Lee, 2006). Females, with lower self-efficacy, were more subject to the unskillful use of e-learning technology than males in China (Ong and Lai, 2006). Compared with males, females in China could increase their self-efficacy dependent on their family support, indicating that e-learning was closely related to social contexts of genders rather than sex itself (Chu, 2010). Motivation could also be explored since it could exert a significant influence on learning strategies (Guo et al., 2021).

Previous studies provided contradictory findings regarding gender differences in e-learning satisfaction. Motivational gender differences were generally not revealed in Malaysia (Marimuthu et al., 2013). No significant gender differences were revealed in the e-learning motivation and satisfaction although e-learning through the mobile platform—Moodle might positively influence e-learning satisfaction and motivation for both males and females in Spain and the UK (Cuadrado-García et al., 2010). No significant effect of gender and age on e-learning readiness or satisfaction was revealed in Hong Kong, China (So and Swatman, 2010). There was no significant gender difference in the e-learning motivation (Yukselturk and Bulut, 2009). There were also other studies reporting no significant gender differences in satisfaction (e.g., Ramírez-Correa et al., 2015) with and attitudes toward the e-learning approach (e.g., Hung et al., 2010) although Hong (2002) argued that gender played an important role in e-learners' satisfaction.

Nevertheless, it was reported that females, planning learning schedules and interacting with instructors more effectively, were more satisfied with e-learning courses than males among mixed participants in Spain and the UK (González-Gómez et al., 2012). Females considered e-learning effective and were thus more satisfied with it than males (Hu and Hui, 2011) although the e-learning motivation of females was significantly lower than that of males (Hu and Hui, 2011). Reverse findings were found by Lu and Chiou (2010) who reported that males were more satisfied with e-learning than females. Social presence in e-learning could improve learners' motivation and satisfaction (Thayalan et al., 2012). Males felt significantly more enjoyable and satisfied with e-learning *via* video models (Hoogerheide et al., 2016).

Previous studies arrived at inconsistent conclusions regarding the gender differences in e-learning performance (Price, 2006; Marimuthu et al., 2013). No gender differences were revealed in e-learning performance (Chen and Tsai, 2007). Gender was also considered an insignificant influencing factor in e-learning performance (Yukselturk and Bulut, 2009). Males' performance was slightly but not significantly higher than females in game-based learning (Chen et al., 2021). However, gender differences were found in the use of technology, e-instruction, technology skillfulness, and information literacy (Aydin, 2011). Besides, social presence in e-learning could decrease the dropout rate (Cobb, 2009) and improve learners' e-learning performance such as critical thinking (Garrison et al., 2000) and online communications (Danchak et al., 2001). E-learning performance was subject to several factors, e.g., motivation and learning strategies, computer competence, perceptions about discussion, critical thinking, peer learning, problem-based learning, interaction, and available help in a Chinese educational context (Zhu et al., 2009).

Gender was, however, not considered a factor that influenced e-learning performance. There was no significant gender difference in language performance, while females showed significantly higher self-efficacy than males (Harb et al., 2014). No gender difference was found in e-learning *via* video modeling examples and both males and females experienced an enhanced self-perceived competence after this e-learning model (Hoogerheide et al., 2016).

Gender differences in attitudes toward e-learning were generally insignificant although there were some different arguments. Students, whether males or females, held positive attitudes toward the e-learning platform—e-HO in China (Lee et al., 2011). Gender did not exert a significant influence on attitudes toward e-learning (Chen and Tsai, 2007). Little evidence was found regarding gender differences in attitudes toward e-learning systems (Albert and Johnson, 2011). However, significant gender differences were reported by some researchers (e.g., Jackson et al., 2001; Shashaani and Khalili, 2001). Males held more positive attitudes (Whitely, 1997) toward e-learning and Chinese learners were more voluntary to access e-learning (Ong and Lai, 2006). Male university students preferred to use e-learning compared with females (Reda and Dennis, 1992). Males held more favorable attitudes toward e-learning than females and the latter held more computer anxiety than the former (Keller et al., 2007) in Sweden and Lithuania. Females held significantly more positive attitudes toward and were more interested in e-learning medical courses with Moodle than males (Harreiter et al., 2011).

However, others found no gender differences in attitudes toward e-learning. They held that the superficial gender differences in attitudes might be caused by different social statuses, economic states, and preferences rather than sex itself (e.g., Bimber, 2000), and gender differences in the attitude were minimized with the rapid popularization of e-technologies and equally easy access to e-learning (Hanauer et al., 2004; Papastergiou and Solomonidou, 2005). For both genders, attitudes toward e-learning were positively correlated with their satisfaction in Cyprus, Thailand, and other countries (Vate-U-Lan, 2020). No significant gender differences among university faculty and students were found in attitudes toward information and communication technology-assisted learning in a university in India (Verma and Dahiya, 2016). Chinese learners' attitude toward the use of e-learning indicated the intention to use e-learning methods (Ong and Lai, 2006). No significant behavioral intention of e-learning was identified between male and female instructors in Jordan (Altawallbeh et al., 2015).

In view of different and even contradictory findings, it is necessary to meta-analytically summarize the gender differences in e-learners' self-efficacy, satisfaction, motivation, attitude, and performance across the world. The research question proposed is “are there any gender differences in e-learners' self-efficacy, satisfaction, motivation, attitude, and performance across the world?”

## Methods

This meta-analysis is implemented based on the Preferred Reporting Items for Systematic Reviews and Meta-Analyses (PRISMA) (Moher et al., 2009). The review board waived the review protocol registry due to the characteristics of this study.

### Eligibility Criteria

The studies will be included if they meet these criteria: (1) They focus on gender differences in e-learning outcomes rather than e-learning technology itself; (2) They are of high quality based on University of West England Framework for Critically Appraising Research Articles (Moule et al., 2003); (3) They adopt a randomized controlled design where a control and experiment group is comparatively analyzed; (4) They can provide enough data for a meta-analysis.

The studies will be excluded if they meet any of these criteria: (1) They focus on e-learning technology itself rather than e-learning outcomes; (2) They study non-human participants; (3) They are written in a language other than English or in the English of academically lower quality; (4) They include participants fewer than nine.

### Data Sources and Search Strategy

To remove duplication of this meta-analysis, the researcher searched multiple databases, e.g., the Cochrane Databases of Systematic Review, the Center for Review and Dissemination, Taylor & Francis Group, Sage Publications, Springer Nature, Web of Science, Science Direct, EBSCO, and Educational Research Complete. To include as comprehensive literature as possible, the researcher considered both published and unpublished literature written in English without time limitations. The researcher included those ranging from their inception to February 10, 2021.

The researcher adopted a three-step search strategy to include studies. Firstly, the researcher selected numerous databases such as Scopus, Taylor & Francis Group, Sage Publications, Springer Nature, Web of Science, Science Direct, Ebsco, Proquest, and Educational Research Complete. Secondly, the researcher comprehensively searched the literature by entering corresponding terms into various databases and obtained results containing a sea of literature. Thirdly, the researcher read through the literature to prevent duplication by optimizing the results.

**Figure 1:**
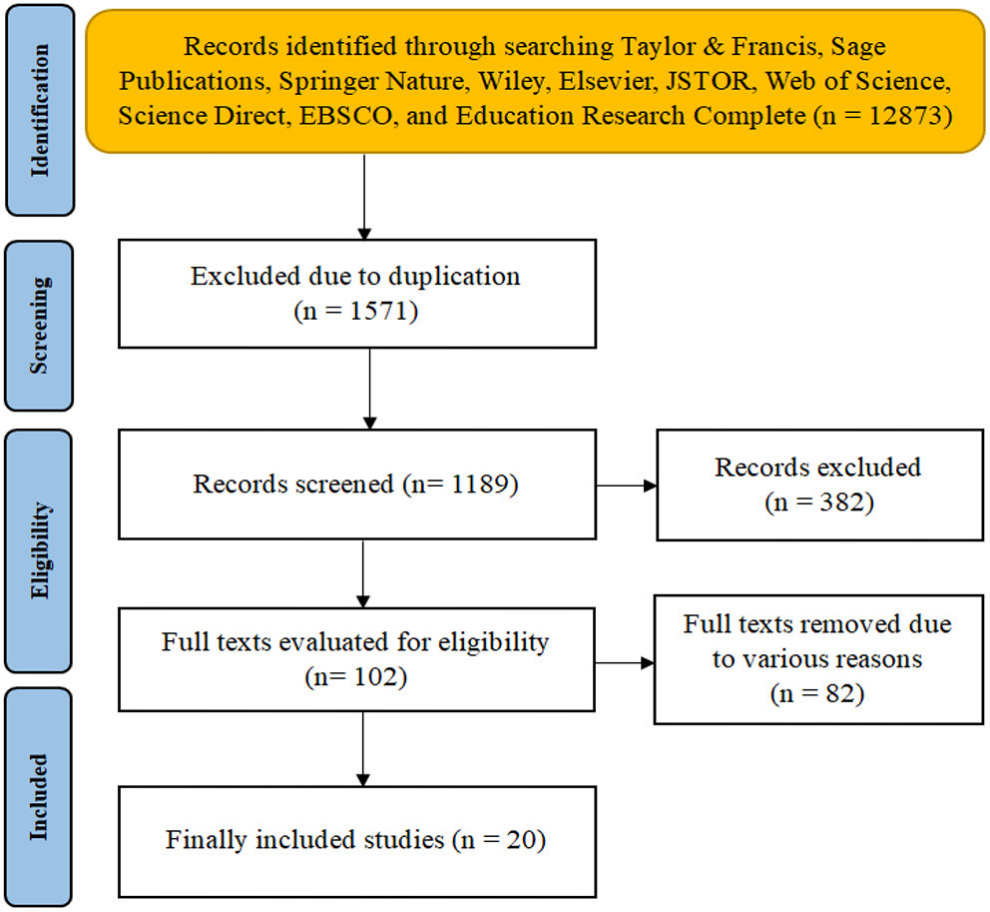
A flow chart of literature inclusion.

The selection process of literature was implemented based on the PRISMA flowchart (Figure 1). Firstly, the obtained results were entered into the software Endnote X8 (Thomson Reuters, New York, USA) for duplication identification and removal. Secondly, two reviewers screened the irrelevant literature by perusing abstracts, keywords, titles, etc. Thirdly, both reviewers independently evaluated the literature for eligibility based on University of West England Framework for Critically Appraising Research Articles (Moule et al., 2003). Fourthly, both reviewers met together to decide the final selection. In case both reviewers could not reach an agreement on any selected literature, a third reviewer would join and determine the selection.

### Quality Assessment

The University of West England Framework for Critically Appraising Research Articles (Moule et al., 2003) evaluated each article in terms of five sections, i.e., *The Introduction, the Methods Section, Ethics, the Results/Findings*, and *the Conclusions*. Each section was evaluated based on a given criterion. For example, as for the introduction part, reviewers evaluated it by proposing criteria such as whether there was a clear statement about the topic being investigated and whether there was a clear rationality for the research. As for the methods section, reviewers evaluated it based on four criteria, i.e., (1) The research design should be clearly described; (2) The research methods should be appropriate for the topic being investigated; (3) The researchers should acknowledge the advantages or disadvantages of the design; (4) There should be a clear statement about how the participants were selected. Each article was be scored based on the criteria. Those top-scored were included in the meta-analysis. The results/findings section required that the results be related to the literature review and the researchers acknowledge the limitations of the research design. In the conclusion section, the researchers should acknowledge the implications for future research, identify areas for further research, and propose recommendations for practice from the results or discussions.

The researcher excluded publications due to various kinds of reasons. The records (*n* = 382) were excluded due to the reasons such as no abstracts (*n* = 7), irrelevance to the educational scope (*n* = 294), non-English publications (*n* = 9), and unconvincing conclusions (*n* = 72). The various reasons for the exclusion of full texts (*n* = 82) included (1) inadequate information for a meta-analysis (*n* = 27), (2) small sample sizes (*n* = 8), (3) lack of rigid design (*n* = 12), (4) editorial collections (*n* = 9), (5) reports (*n* = 12), and (6) irrelevance to the research focus (*n* = 14).

### Data Extraction

Both reviewers extracted specific data from the included studies. The extracted data included total numbers of participants, means, and standard deviations in both control and experimental groups, levels of education of participants, modes of e-learning, countries where the study was conducted, e-learning outcomes (e-learners' attitudes, motivation, performance, satisfaction, and self-efficacy), and data collection methods. In case the data were not enough for the meta-analysis, the researcher would correspond with the authors. The study would be removed if the researcher finally failed to obtain enough data for the meta-analysis. The main extracted data are shown in Table 1.

**Table 1:** Characteristics of included studies.

***N***	**References**	**Outcome**	**Data collection**	**Country**	**participant**
1	Albert and Johnson (2011)	Satisfaction	Survey	USA	University students
2	Altawallbeh et al. (2015)	Attitude	Survey	Jordan	University students
3	Baylor and Kim (2004)	Self-efficacy	Test	USA	University students
4	Chu (2010)	Self-efficacy	Survey	China	Community college and senior learning center staff
5	Cuadrado-García et al. (2010)	Satisfaction	Moodle platform	mixed	University students
6	González-Gómez et al. (2012)	Satisfaction	Moodle platform	mixed	University students
7	Harb et al. (2014)	Self-efficacy	Test	Jordan	University students
8	Harreiter et al. (2011)	Attitude	Survey	Austria	University students
9	Hoogerheide et al. (2016)	Satisfaction	Test	The Netherlands	Secondary school students
10	Hu and Hui (2011)	Self-efficacy	Test	China	University students
11	Lee et al. (2011)	Attitude	e-HO platform	China	University students
12	Marimuthu et al. (2013)	Performance	Survey	Malaysia	University students
13	Ong and Lai (2006)	Self-efficacy	Survey	China	Company staff
14	Ramírez-Correa et al. (2015)	Performance	Survey	Chile/Spain	University students
15	So and Swatman (2010)	Satisfaction	Survey	China	Primary and secondary school in-service teachers
16	Thayalan et al. (2012)	Motivation	Survey	Indonesia	University students
17	Tung and Deng (2007)	Motivation	Survey	China	Elementary school students
18	Vate-U-Lan (2020)	Satisfaction	Survey	Mixed	University/secondary students
19	Verma and Dahiya (2016)	Attitude	Survey	India	University students
20	Zhu et al. (2009)	Satisfaction	Test	China	University students

### Statistical Analysis

The researcher conducted the meta-analysis generally through Stata MP/14.0. Specifically, the researcher entered related data into Stata MP/14.0 to calculate standardized mean differences (SMD) or Cohen *d*, the lower and upper bounds of 95% confidence intervals, weights, distribution of individual studies, Q data, heterogeneity, I-squared (I^2^), *p* values, and pooled results, which was presented by forest plots. Cohen *d* is calculated as the mean difference between the experimental and control group divided by the standard deviation of the learning outcome across both groups (Sedgwick and Marston, 2013).

The statistics I^2^, calculated as the percentage of the total variation of all included studies, was used to measure the heterogeneity of effect sizes. The heterogeneity was considered commonly existent in different studies. Thus, the researcher measured it through Higgins and Green's criteria (Higgins and Green, 2011), i.e., the heterogeneity would be considered unimportant if 0% < I^2^ <40%, moderate if 30% < I^2^ <60%, substantial if 50% < I^2^ <90%, and considerable if 75% < I^2^ <100%. If I^2^ was larger than 50%, the results would prove significantly heterogeneous. The researcher would then adopt a random-effect model to conduct the meta-analysis. If I^2^ was smaller than 50%, the results would prove insignificantly heterogeneous. The researcher would thus conduct the meta-analysis using a fixed-effect model.

Z statistics was adopted to test the publication bias. The *p-*value being smaller than 0.05 indicated the presence of the publication bias while its being larger than 0.05 indicated the absence of the publication bias. The researcher also tested the publication bias *via* Begg's and Egger's tests through funnel plots where no-effect lines and individual studies were shown, as well as specific effect sizes and standard errors of effect sizes. The symmetric distribution of dots along the no-effect line in a funnel plot indicated the absence of the publication bias while the asymmetric distribution indicated the presence of the publication bias.

## Results

### Study Selection

According to the PRISMA flowchart (Moher et al., 2009), the researcher obtained a total of 12,873 results from several databases, i.e., Taylor & Francis, Sage Publications, Springer Nature, Wiley, Elsevier, JSTOR, Web of Science, Science Direct, EBSCO, and Educational Research Complete. The researcher obtained 1,571 results after removing 11,302 duplicated results *via* Endnote. Two reviewers selected 1,189 results after independently screening and excluding 382 results after perusing abstracts, titles, and keywords. A total of 102 results passed the evaluation process. After removing 82 results due to various reasons such as incomplete data, improper design, and missing information, the researcher selected 20 full texts. The researcher then undertook the meta-analysis based on the included 20 studies, whose major characteristics were summarized in Table 1.

### Characteristics of Studies

As shown in Table 1, the researcher summarized the main characteristics of included studies. The studies were conducted in various countries across the world, e.g., China, the USA, Austria, the Netherlands, Jordan, Chile, Spain, Malaysia, Indonesia, the UK, and India. The e-learning modes included a single e-learning course, multiple e-learning courses, inter-disciplinary e-learning courses, and various e-learning platforms. The educational levels of participants included university, elementary and secondary schools, and community college. The data collection methods included surveys, pre- and post-tests, a written final assessment test, e-learning platforms such as e-HO, Moodle, and online English tests. The e-learning outcomes were classified into satisfaction, attitude, motivation, self-efficacy, and performance. The included studies could be classified into peer-reviewed journal articles, conference articles, and book chapters.

### Tests of Publication Bias

**Figure 2:**
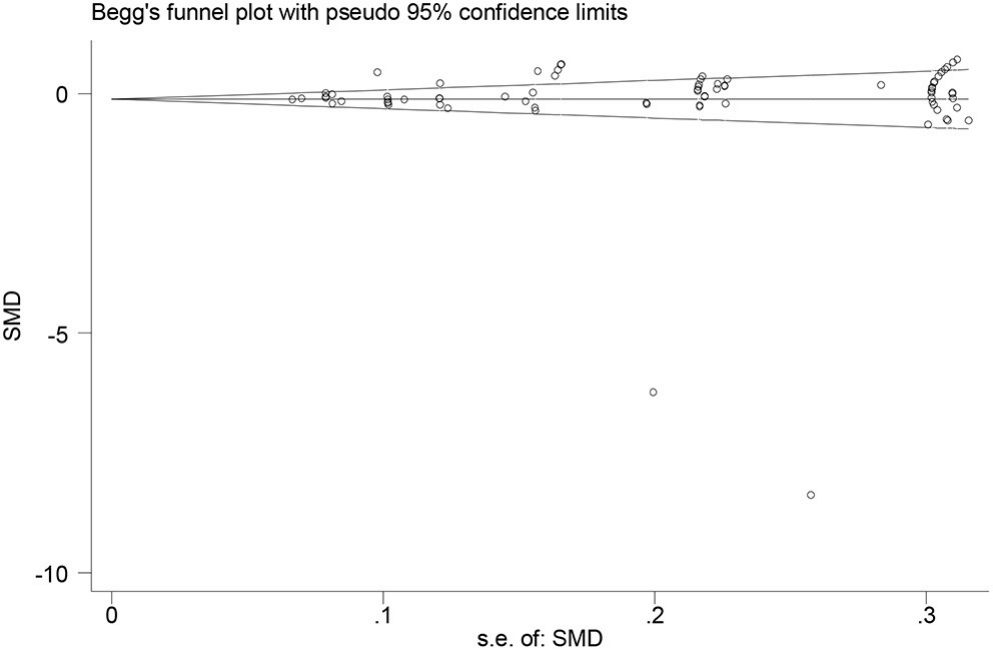
A funnel plot of publication bias using Begg's test.

To enhance the reliability of the results, the researcher tests the publication bias using both Begg's and Egger's tests. As for Begg's test, the researcher tests the publication bias using “metabias _ES _seES, begg” as a command to test the rank correlation between standardized intervention effect and its standard error (data input format theta se_theta assumed). The results indicate the absence of publication bias [Kendall's Score (P-Q) = 144, Std. Dev. of Score = 227.36, z = 0.63, Pr > |z| = 0.529]. As shown in Figure 2, a dot indicates an individual study. The dots are distributed along both sides of the middle line non-asymmetrically, indicating the absence of publication bias.

**Figure 3:**
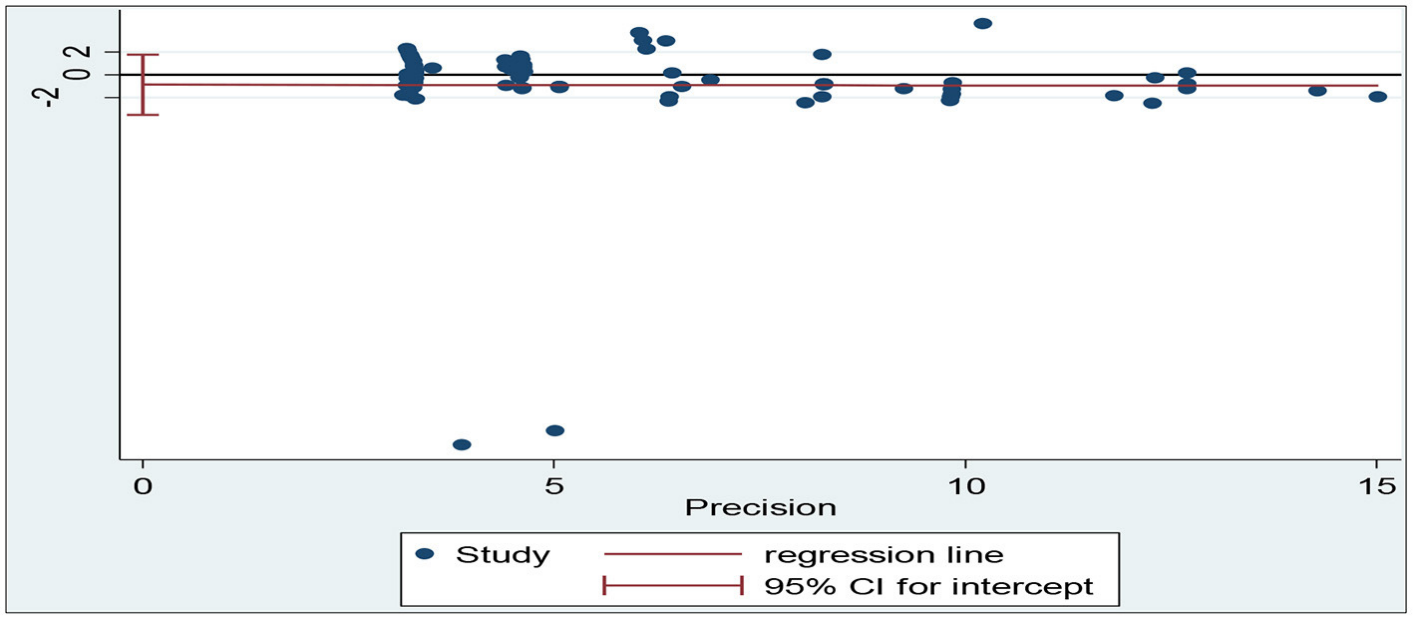
A plot of publication bias using Egger's test.

As for Egger's test, the researcher enters the command “metabias _ES _seES, egger graph” into Stata MP/14.0 for detection of the publication bias since Egger's test can detect publication bias more sensitively than Begg's test (Egger et al., 1997). It is shown in Figure 3 that the studies are nearly symmetrically distributed along both sides of the regression line. The researcher therefore concludes that the results indicate the absence of publication bias (t = −0.64, *p* = 0.523, 95% confidence interval = −3.49–1.79).

### A Sensitivity Analysis

**Figure 4:**
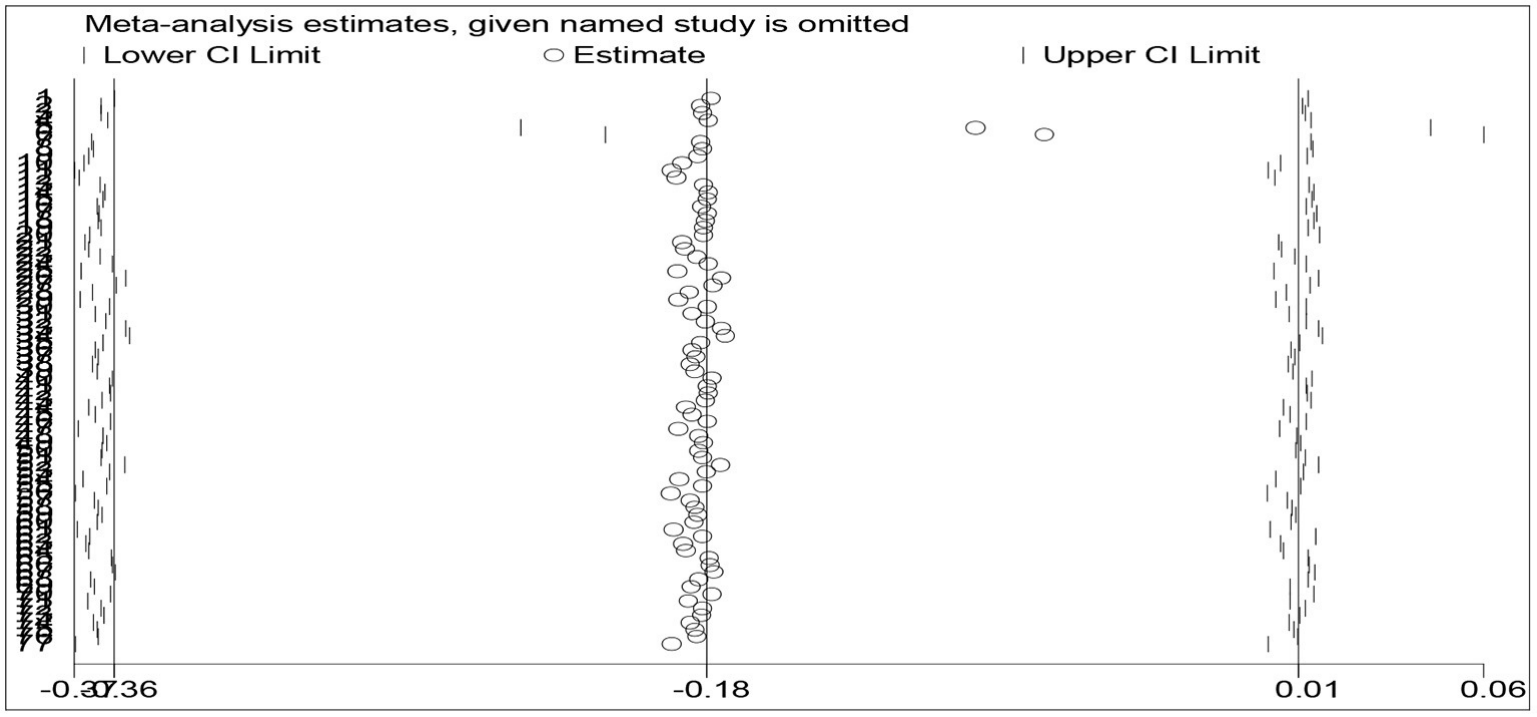
A plot of sensitivity analysis.

The sensitivity analysis is used to test the reliability or robustness of the meta-analysis *via* a leave-one-out method. If the leave-one-out method produces consistent results, then the meta-analysis will be considered robust or reliable. To conduct the sensitivity analysis, the researcher enters “numbers of participants, means, and standard deviations” across both experimental and control groups for the *metan-based* influence analysis. As shown in Figure 4, the meta-analysis estimates are all positioned between the upper and lower bounds of the 95% confidence interval if a study is omitted. The researcher, therefore, conclude that the meta-analysis results are robust or reliable.

### Gender Differences in E-Learners' Self-Efficacy in Different Countries

**Figure 5:**
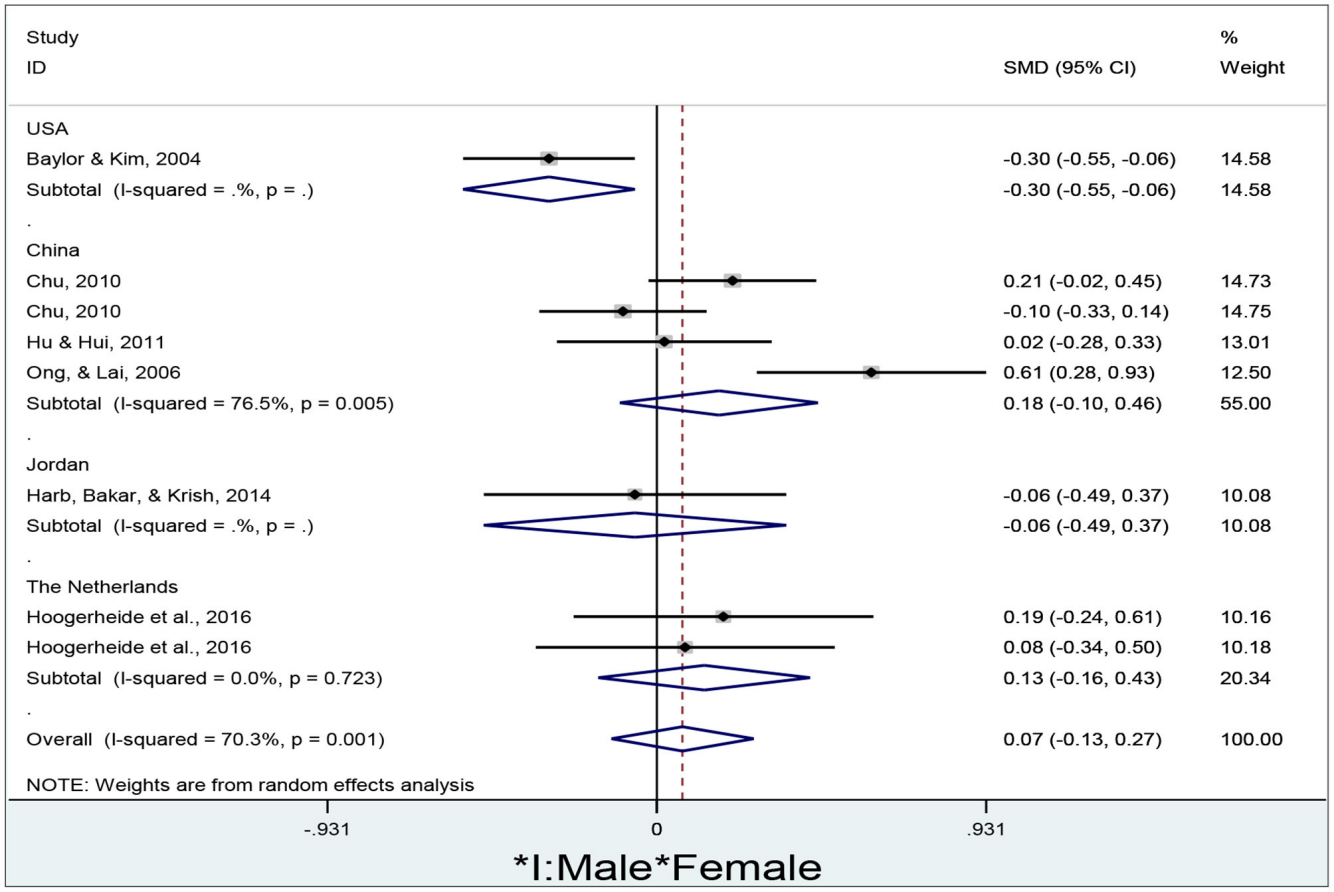
Gender differences in e-learners' self-efficacy in different countries.

To determine whether a random-effect or fixed-effect model was used to run the meta-analysis of gender differences in e-learners' self-efficacy in different countries, the researcher firstly tested the heterogeneity of the meta-analysis estimates *via* a forest plot through Stata/MP 14.0 (Figure 5).

As shown in Figure 5, the researcher obtains a total of 8 effect sizes to determine gender differences in e-learners' self-efficacy in different countries such as the USA, China, Jordan, and the Netherlands. Since the overall results are significantly heterogeneous (I^2^ = 70.3, *p* = 0.001), the researcher adopts a random-effect model to conduct the meta-analysis. The diamond indicates the pooled effect of e-learners' self-efficacy between males and females in different countries. In the USA, females present significantly higher self-efficacy than males (d = −0.30, 95% CI = −0.55 to −0.06, z = 2.46, *p* = 0.014) since the diamond is located to the left of the no-effect line. However, no significant gender differences in e-learners' self-efficacy are shown in China (d = 0.18, 95% CI = −0.10–0.46, z = 1.23, *p* = 0.219), Jordan (d = −0.06, 95% CI = −0.49–0.37, z = 0.28, *p* = 0.778), the Netherlands (d = 0.13, 95% CI = −0.16–0.43, z = 0.88, *p* = 0.379), and overall results (d = 0.07, 95% CI = −0.13–0.27, z = 0.71, *p* = 0.478) since the diamonds all cross the no-effect line.

### Gender Differences in E-Learners' Satisfaction in Different Countries

**Figure 6:**
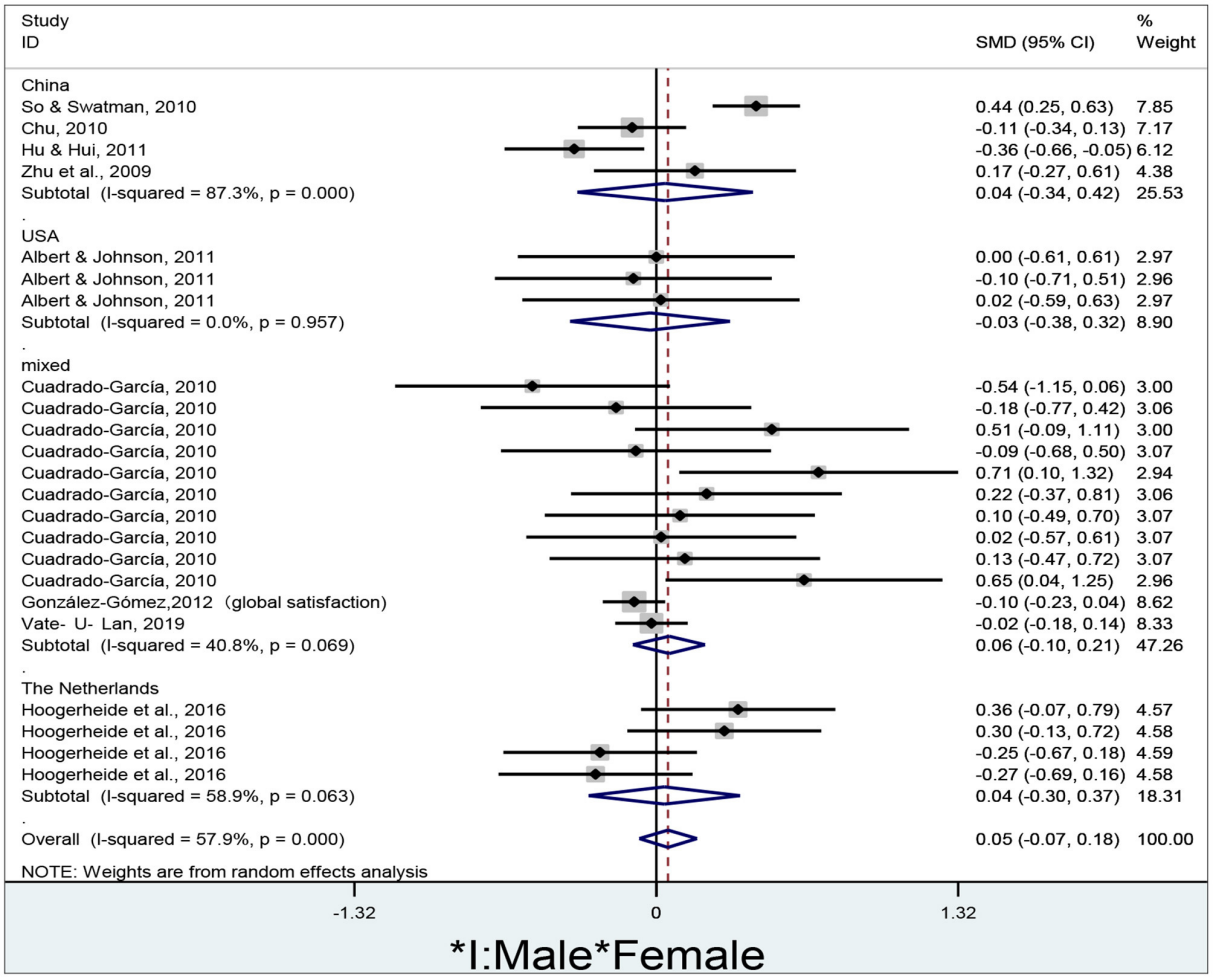
Gender differences in e-learners' satisfaction in different countries.

To summarize gender differences in e-learners' satisfaction in different countries, the researcher draws a forest plot using Stata/MP 14.0 (Figure 6).

The researcher obtains a total of 23 effect sizes to determine gender differences in e-learners' satisfaction in different countries. The researcher adopts a random-effect model to conduct the meta-analysis since the overall estimates are significantly heterogeneous (I^2^ = 57.9%, *p* < 0.01). No significant gender differences in e-learners' satisfaction are revealed in China (d = 0.04, 95% CI = −0.34–0.42, z = 0.20, *p* = 0.842), the USA (d = −0.03, 95% CI = −0.38–0.32, z = 0.15, *p* = 0.882), mixed countries (d = 0.06, 95% CI = −0.10–0.21, z = 0.70, *p* = 0.484), the Netherlands (d = 0.04, 95% CI = −0.30–0.37, z = 0.21, *p* = 0.832), and overall results (d = 0.05, 95% CI = −0.07–0.18, z = 0.81, *p* = 0.421) since all of their diamonds cross the no-effect line.

### Gender Differences in E-Learners' Motivation in Different Countries

**Figure 7:**
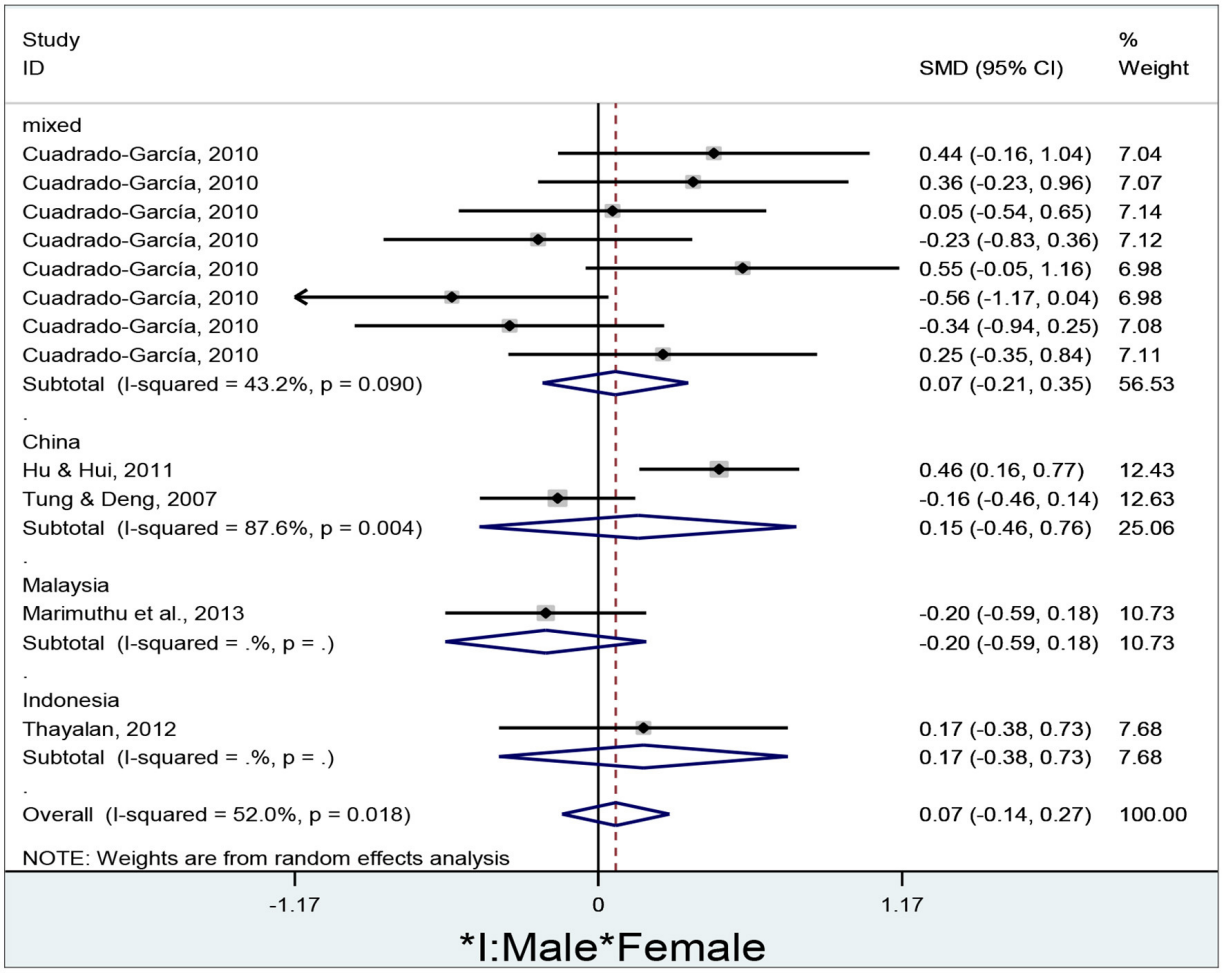
Gender differences in e-learners' motivation in different countries.

To examine the pooled effect of gender differences in e-learners' motivation in different countries, the researcher draws a forest plot using Stata/MP 14.0 (Figure 7).

The researcher obtains a total of 12 effect sizes to examine e-learners' motivation in different countries. Since the overall results are significantly heterogeneous (I^2^ = 52.0%, *p* = 0.018), the researcher adopts a random-effect model to conduct the meta-analysis. No significant gender differences are found in e-learners' motivation in mixed countries (d = 0.07, 95% CI = −0.21–0.35, z = 0.46, *p* = 0.645), China (d = 0.15, 95% CI = −0.46–0.76, z = 0.49, *p* = 0.623), Malaysia (d = −0.20, 95% CI = −0.59–0.18, z = 1.02, *p* = 0.306), Indonesia (d = 0.17, 95% CI = −0.38–0.73, z = 0.61, *p* = 0.540), and overall results (d = 0.07, 95% CI = −0.14–0.27, z = 0.63, *p* = 0.527) since all of their diamonds cross the no-effect middle line.

### Gender Differences in E-Learners' Attitude in Different Countries

**Figure 8:**
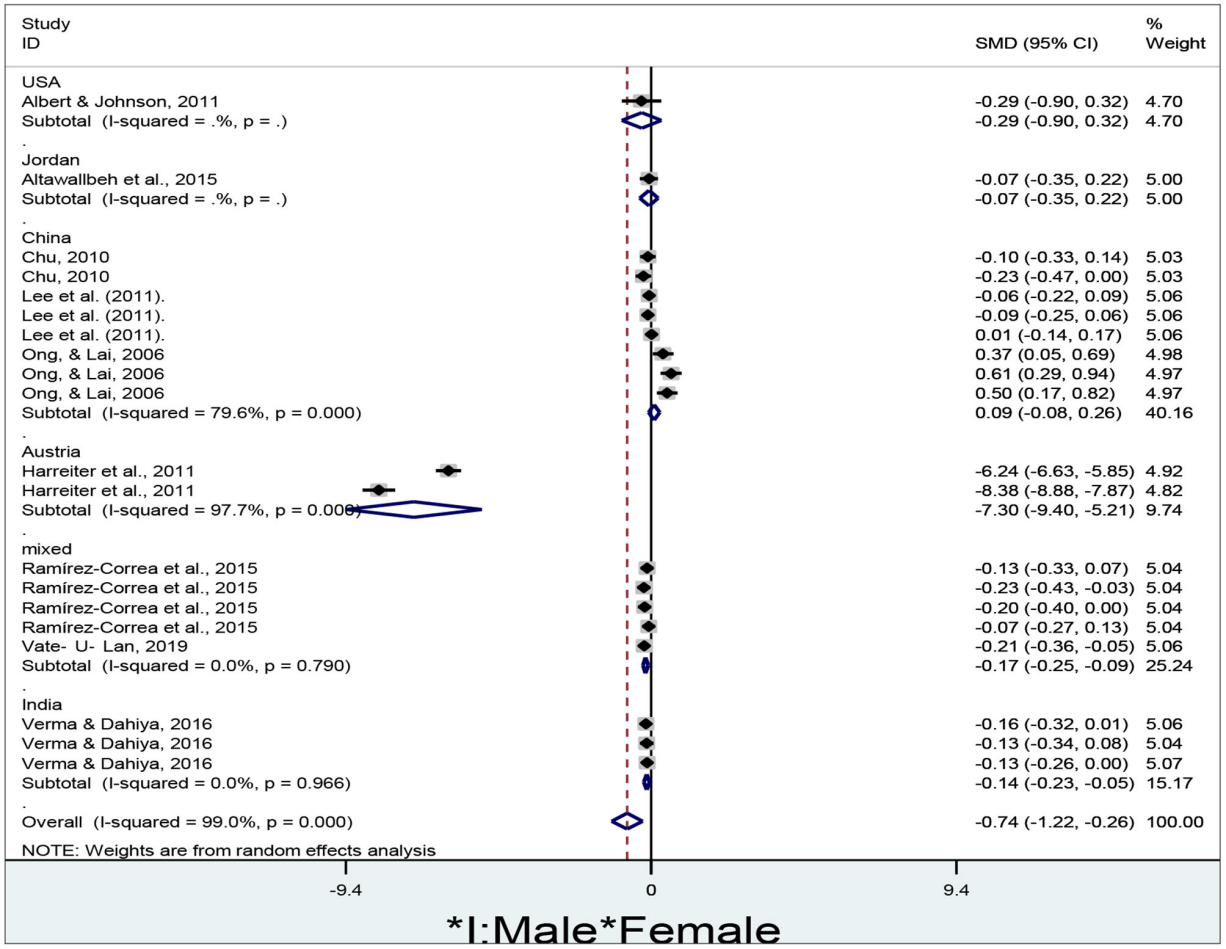
Gender differences in e-learners' attitude in different countries.

To examine gender differences in e-learners' attitude in different countries, the researcher drew a forest plot using Stata MP 14.0 (Figure 8).

The researcher obtained a total of 20 effect sizes to summarize the gender differences in e-learners' attitude in different countries. A random-effect model was adopted to run the meta-analysis since the overall results are significantly heterogeneous (I^2^ = 99%, *p* < 0.01). No significant gender differences in e-learners' attitudes are found in the USA (d = −0.29, 95% CI = −0.90–0.32, z = 0.94, *p* = 0.346), Jordan (d = −0.07, 95% CI = −0.35–0.22, z = 0.45, *p* = 0.65), and China (d = 0.09, 95% CI = −0.08–0.26, z = 1.05, *p* = 0.292) since their diamonds all cross the no-effect middle line. However, females' attitudes are significantly higher than males' in Austria (d = −7.30, 95% CI = −9.40 to −5.21, z = 6.83, *p* < 0.01), India (d = −0.14, 95% CI = −0.23 to −0.05, z = 2.92, *p* = 0.004), mixed countries (d = −0.17, 95% CI = −0.25 to −0.09, z = 3.94, *p* < 0.01), and overall results (d = −0.74, 95% CI = −1.22 to −0.26, z = 3.04, *p* = 0.002) since all of their diamonds are located to the left of the no-effect middle line.

### Gender Differences in E-Learners' Performance in Different Countries

**Figure 9:**
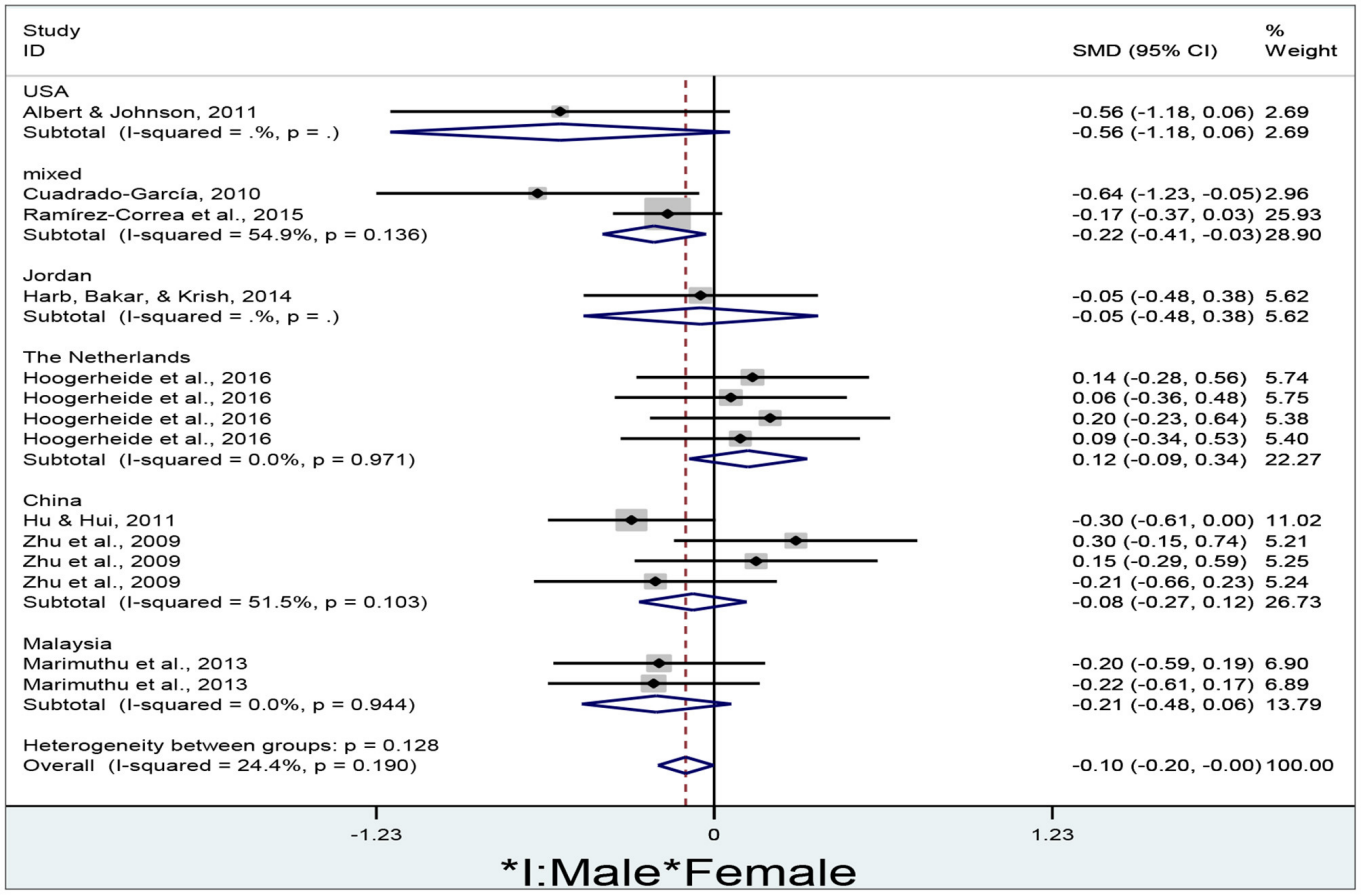
Gender differences in e-learners' performance in different countries.

The researcher obtained a total of 14 effect sizes to determine gender differences in e-learners' performance in different countries (Figure 9).

The researcher adopted a fixed-effect model to conduct the meta-analysis since the overall results are not significantly heterogeneous (I^2^ = 24.4%, *p* = 0.19). No significant gender differences in e-learners' performance are revealed in the USA (d = −0.56, 95% CI = −1.18–0.06, z = 1.78, *p* = 0.075), Jordan (d = −0.05, 95% CI = −0.48–0.38, z = 0.23, *p* = 0.822), the Netherlands (d = 0.12, 95% CI = −0.09–0.34, z = 1.13, *p* = 0.259), China (d = −0.08, 95% CI = −0.27–0.12, z = 0.78, *p* = 0.435), and Malaysia (d = −0.21, 95% CI = −0.48–0.06, z = 1.51, *p* = 0.131) since their diamonds all cross the no-effect middle line. However, female performance is significantly higher than male in mixed countries (d = −0.22, 95% CI = −0.41 to −0.03, z = 2.27, *p* = 0.023), and overall results (d = −0.10, 95% CI = −0.20–0.00, z = 2.00, *p* = 0.046) since their diamonds are located to the left of the no-effect middle line.

## Discussion

The findings of this study are generally consistent with previous research (e.g., Bimber, 2000; Baylor and Kim, 2004; Pituch and Lee, 2006; Chen and Tsai, 2007; Yukselturk and Bulut, 2009; González-Gómez et al., 2012; Marimuthu et al., 2013). As for e-learners' self-efficacy, no significant gender differences have been revealed in all of the countries except the USA. Baylor and Kim's study (2004), conducted in the USA, concluded that females had significantly higher self-efficacy than males in the e-learning context. Female agents (around 61%) greatly outnumbered males (around 39%), which might have caused gender bias. The agents, merely representing gender-specific features, might have led to results different from the real human participants although agents did play an important role in e-learning experiments. Participants working with female agents might have been positively influenced by their soft, encouraging voice and image, followed by enhanced self-efficacy.

The researcher did not find any significant gender difference in e-learners' satisfaction in different countries. E-learning, as an innovative learning method, drew many learners' attention whether they were biologically male or female. It could bring great convenience to them through the advanced information technologies. Learners did not need to carry any heavy learning materials with them and they could engage in learning wherever and whenever they wanted to. Through e-learning platforms, they could swiftly transfer a huge amount of data and easily had access to learning resources. They could also enhance their satisfaction with e-learning through frequent interactions with peers or teachers to solve difficult problems and arrange their learning activities. Teachers could gather enough data regarding students' feedback and decide teaching progress accordingly. This could improve both teachers' and students' satisfaction with the information technology-assisted pedagogical approach.

No significant gender differences in motivation were revealed among e-learning participants. In the e-learning environment, learners could manage their learning activities on their own. E-learning activities were no longer limited by the physical classroom and the face-to-face teacher. They could establish learning goals, select learning contents, and determine learning styles based on their own preferences. E-learning provided unprecedented learning resources and created an innovative learning environment, where learners were greatly motivated to join the learning activities since they could conveniently learn *via* various kinds of apps, texts, videos, audios, and technologies. The e-learning environment also bridged the gap of communication through online collaborations. Learners could seek help from peers and resort to teachers for enquiry of difficult questions at will. They could also determine the learning progress and styles based on their own preferences, rather than limited to a certain style or progress. In this way, their learning motivation was improved whether they were female or male.

In the USA, Jordan, and China, there were no significant gender differences in the attitudes toward e-learning. Since both genders held positive attitudes toward e-learning, designers and teachers might not need to cater the e-learning approach to a specific gender but to other demographics such as economic status (Albert and Johnson, 2011). When designing the e-learning strategy, teachers could comprehensively consider the age and experience of Internet use to popularize and improve the effectiveness of the use of e-learning approaches (Altawallbeh et al., 2015). Although no significant gender differences in attitudes were found toward e-learning, both genders held lower levels of communication self-efficacy (Chu and Tsai, 2009). Communication skills, different from simple clicking, surfing, or glimpsing, might need complicated cognitive involvements such as coordination of finger and eye movements and mental processing (Chu, 2010).

However, in Austria, India, and mixed countries (Chile and Spain), females held significantly more positive attitudes toward e-learning than males. Females might join or initiate more communications with peers and teachers, hold more social presence, and thus feel more satisfied with e-learning activities, followed by more positive attitudes than males who sought information rather than communication using the Internet (Johnson, 2011; González-Gómez et al., 2012). Males, mostly aiming at personal success and higher social status, were isolated from their peers and involved in critical thinking although psychological researchers proved no gender differences in their mental inborn feedback to surroundings (Salomone, 2007). The e-learning platform could provide learners with a large number of resources and opportunities, where females showed significantly more intense interest in gender issues which were criticized by males (Harreiter et al., 2011). Females might spend more time examining contents through the e-learning approach, leading to more positive attitudes than their male counterparts.

In general, females more positively evaluate e-learning than males since the pooled diamond is situated to the left of the no-effect line (Figure 8). Submerged in abundant information in the e-learning platform, females could be more interested in their favorite issues such as gender-related learning materials while males aimed to seek information beneficial to their purpose. Females might concentrate more on the interesting issues than males who aimed to seek information that could improve their social status. Concerning learning issues, females might show more interest than males since the former aimed at gender-based learning issues and acquired knowledge through communication and social presence while the latter aimed at social rank issues (Harreiter et al., 2011). Males were distracted by a sea of information in case they could not find the information they needed. In the e-learning context, males were more likely to present personal information representing their social status, while females were more likely to enjoy the benefits of social networking when social information was reduced. Females paid more attention to learning and social process and less attention to members of a learning community than males (Flanagin et al., 2002). This might enhance female attitudes toward e-learning and reduce male positive evaluation of an e-learning method.

Significant gender differences in e-learning performance were found among students at the London School of Economics (the UK) and University of Valencia (Spain) (Cuadrado-García et al., 2010). Females significantly outperformed males. As the authors mentioned, females greatly outnumbered males, which might have caused bias in results. The researcher failed to reveal any gender difference in e-learners' performance in other countries such as the USA, the Netherlands, Jordan, Malaysia, and China. The new decade has been witnessing the dramatic development of information technologies. Both males and females nowadays have equally convenient access to e-learning approaches in most of the countries across the world. Both genders performed similarly but in the e-learning process, males paid more attention to the competitiveness in the course, while females regarded the virtual classroom as an opportunity for online cooperative learning and cherished the cooperative e-learning environment (Arbaugh, 2002). Different preferences might have offset their different performance levels and caused insignificant gender differences in e-learning performance.

The e-learning environment could greatly facilitate discussion and opinion sharing, which could promote efficient information exchange and cultivate social relations between males and females (Wang et al., 2007). Social constructivists (e.g., Derry et al., 2000) argued that discussion and opinion sharing could help learners construct high-quality knowledge structures. Through an appropriate teaching design, teachers could encourage students to solve difficult problems and facilitate active debates by gathering them online. Through frequent interactions and intentional organization of the teacher, balanced numbers of males and females could form an effective learning community under the supervision and guidance of the teacher, where both males and females could mutually assist for knowledge acquisition. Discussion and opinion sharing could bridge the gap of communication between males and females. They could increase their knowledge and improve their social skills, conducive to favorable e-learning performance. Different characteristics of both genders might have offset the originally different performance levels through the interactive process in the e-learning process.

## Conclusion

### Major Findings

This study, including 20 high-quality publications, meta-analytically examined gender differences in e-learning outcomes, e.g., e-learners' self-efficacy, satisfaction, motivation, attitude, and performance across the world. Generally, there are no significant gender differences in e-learning learning outcomes. Specifically, exceptions are that females significantly outperformed males in Spain and the UK, that in Austria, India, and mixed countries (Chile and Spain), females hold significantly more positive attitudes toward e-learning than males, and that in the USA, females present significantly higher self-efficacy than males. The popularity of information technologies among males and females may have played an important role in minimizing gender differences in e-learning outcomes.

## Limitations

While this study is rigidly designed based on the PRISMA flow process, there are still several limitations. Firstly, this study merely includes publications written in English, which may have caused publication bias. Secondly, this study cannot include all of the literature due to the limitation of the library resources. For instance, we did not obtain the data from MDPI, Frontiers, Dove Press, preprint servers, PubMed, etc. Thirdly, the included studies may have biases themselves, which may have caused bias in results. Among the 20 included studies, 14 studies are solely on university students. This may indicate the potential bias of the included studies.

### Future Research Directions

Future research may adopt other methods to identify gender differences in the e-learning environments except for a meta-analytical review. The gender-sensitive method in sentimental analysis can also be considered to study gender differences in e-learning since it can identify gender differences by providing immediate information of emotions (Usart et al., 2022). The analysis of posts in an online discussion forum is also a reliable method to provide plentiful resources for the research into gender differences in e-learning since it is a frequently used tool to transmit information and provide peer comments (Ogange et al., 2018). A Unified Theory of Acceptance and Use Technology model can be constructed to study gender differences in e-learning to provide references for policy makers and course designers (Alghamdi et al., 2022).

In the future, gender differences in e-learning can be examined *via* interdisciplinary cooperation such as sociology and computation. During and after the COVID-19 pandemic, future research into e-learning will be conducive to social equity and development. Future research could focus on how to provide high-quality support for the male e-learners (Noroozi et al., 2022) to improve social equity, especially in the countries where female e-learners outperform males. Gender differences and preferences can be seriously considered when multimedia technology is adopted in the e-learning process (Wang and Hung, 2022), which needs the cooperation of the computation field. In the future, more digital tools can be developed and designed to transform the traditional learning to e-learning and to bridge the digital gender gap in the e-learning era (Palomares-Ruiz et al., 2020).

Future research can also investigate the factors that may be under the influence of gender differences in e-learning. Students' perceived personalized learning support, academic achievement, and behavioral intention may significantly be influenced by gender differences in e-learning (Wongwatkit et al., 2020). E-learning designers can pay enough attention to this finding and take effective measures to minimize this gender effect. Motivation and academic achievements can more significant influence girls than boys (Hermes et al., 2021). Teachers can adopt different teaching strategies to motivate different genders. Future research can extend and leverage the effects of gender differences to maximize the e-learning effectiveness and efficiency.

## Data Availability Statement

The original contributions presented in the study are included in the article/Supplementary Material, further inquiries can be directed to the corresponding author.

## Author Contributions

ZY: conceptualization, design, writing, editing, and analysis. XD: writing, editing, and analysis. All authors contributed to the article and approved the submitted version.

## Conflict of Interest

The authors declare that the research was conducted in the absence of any commercial or financial relationships that could be construed as a potential conflict of interest.

## Publisher's Note

All claims expressed in this article are solely those of the authors and do not necessarily represent those of their affiliated organizations, or those of the publisher, the editors and the reviewers. Any product that may be evaluated in this article, or claim that may be made by its manufacturer, is not guaranteed or endorsed by the publisher.
